# Acute cricopharyngeal achalasia after general anesthesia in myotonic dystrophy: A case report

**DOI:** 10.1097/MD.0000000000036378

**Published:** 2023-12-01

**Authors:** Yunjung Rho, Jinmann Chon, Myung Chul Yoo, Ga Yang Shim, Sung Joon Chung, Yunsoo Soh

**Affiliations:** a Department of Physical Medicine and Rehabilitation Medicine, Kyung Hee University Hospital, Seoul, Republic of Korea; b Department of Physical Medicine and Rehabilitation, Kyung Hee University Hospital at Gangdong, Seoul, Republic of Korea.

**Keywords:** cricopharyngeal achalasia, general anesthesia, myotonic dystrophy type 1, upper, upper esophageal sphincter, videofluoroscopic swallowing study

## Abstract

**Rationale:**

Myotonic dystrophy type 1 (DM-1) is a progressive multisystem genetic disorder that causes myotonia and both distal limb and facial/neck muscle weakness by expanding the CTG repeats of the DMPK gene in chromosome 19q13.3. General anesthesia is indicated in DM-1 patients owing to their sensitivity to anesthetic drugs such as opioids, hypnotics, and neuromuscular blocking agents.

**Patient concerns:**

A 48-year-old male patient underwent a laparoscopic cholecystectomy for gallstones under general anesthesia. He experienced sudden cardiac arrest and respiratory failure the day after surgery. After a thorough review of past medical history, we recognized that 15 years prior, he had been diagnosed with classic type DM-1, but the diagnosis was not self-reported before general anesthesia. Symptoms of severe dysphagia developed subsequently. In a videofluoroscopic swallowing study (VFSS), we observed abrupt aggravation of myotonic dysphagia after general anesthesia. VFSS revealed cricopharyngeal opening dysfunction, with a remaining large residue in the pyriform sinus, resulting in a severe cricopharyngeal achalasia pattern.

**Diagnosis:**

Acute cricopharyngeal achalasia after general anesthesia.

**Intervention and outcome:**

The patient underwent a dysphagia rehabilitation program that included cricopharyngeal opening exercises and functional electrical stimulation. However, no significant improvement was observed in the cricopharyngeal achalasia in a 3-month follow-up VFSS.

**Lessons:**

Low body temperature and anesthetic medications such as opioids and hypnotic agents can induce myotonia in the cricopharyngeal muscle.

## 1. Introduction

Myotonic dystrophy type 1 (DM-1) is an autosomal dominant, progressive, multisystemic genetic disorder that mainly causes myotonia, distal limb and facial/neck muscle weakness, cardiac arrhythmia, and cataracts.^[1]^ The prevalence of DM-1 varies between 2.1 and 14.3 per 100,000 people and is less common in Asia.^[1]^ DM-1 is caused by the expansion of CTG repeats of the DMPK gene in chromosome 19q13.3. A CTG length exceeding 34 repeats is considered abnormal. General anesthesia (GA) in DM-1 patients can affect the physiological features of the disease.^[1]^ DM-1 has an increased sensitivity to drugs used for anesthesia, such as opioids, hypnotics, and neuromuscular blockers. Low body temperature and anesthetic medications can cause tremors, resulting in myotonia. Cardiorespiratory, musculoskeletal, and myotonic aggravation after GA has been reported.^[2,3]^ Herein, we report the case of a DM-1 patient who suffered abrupt aggravation of cricopharyngeal achalasia after GA.

## 2. Case presentation

**Figure 1.**
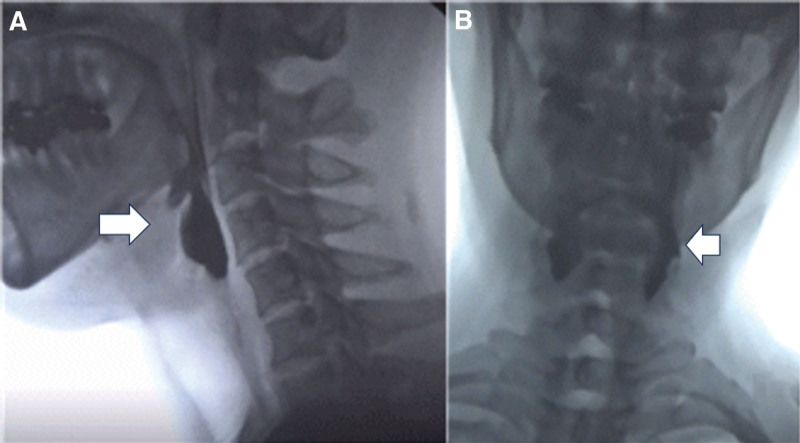
Still image during a videofluoroscopic swallowing study (VFSS). The VFSS reveals cricopharyngeal opening dysfunction during pharyngeal phase. Notely, even though the larynx was elevated and the epiglottis blocked the trachea, the upper esophageal sphincter failed to relax. (A) screenshot showing the lateral view; (B) screenshot of the anterior–posterior view showed a large amount of residue in both pyriform sinuses.

A 48-year-old Asian male patient (weight: 72 kg, height: 171 cm) underwent laparoscopic cholecystectomy for a 1.5-cm gallbladder stone under GA. His general health condition was good, except for a medical history of cured tuberculosis 30 years prior. No specific findings in the cardiac or pulmonary systems were observed preoperatively. Perioperatively, anesthetic agents, including neuromuscular blockers, sedative-hypnotics, and halogenated inhalants were administered. The patient was administered intravenous propofol (120 mg), followed by rocuronium (50 mg) and remifentanil (1 mg) mixed with 100 mL of normal saline. At the end of the surgery, sugammadex (200 mg) was administered to reverse muscle relaxation. The operation concluded without complications, and adequate anesthetic recovery was achieved. However, the patient experienced sudden cardiac arrest and respiratory failure the day after surgery. Electrocardiography revealed ventricular tachycardia with atrial flutter, but a chest radiograph revealed normal findings. Proximal limb weakness and severe dysphagia were observed after the event. The patient complained of pharyngeal muscle weakness and residue. He also had symptoms of heartburn and gastroesophageal regurgitation. After a thorough review of the patient’s medical history, we discovered that 15 years prior, he had been diagnosed with classic DM-1, with an expansion of 580 CTG repeats in the DMPK gene. The patient’s symptoms were not severe; therefore, he did not report his DM-1 history in the preoperative assessment. Although the patient had no corresponding family history, he showed other typical features of the disease, including frontal baldness, distal limb atrophy, cardiac arrhythmia, gallbladder stones, and cataracts, but no dysphagia. After a videofluoroscopic swallowing study (VFSS), we observed the abrupt presence of myotonic cricopharyngeal achalasia. VFSS indicated cricopharyngeal opening dysfunction, the remaining large residue in the pyriform sinus, and even though the larynx was elevated and the epiglottis blocked the trachea, the upper esophageal sphincter failed to relax (Fig. 1). The dysphagia rehabilitation program included cricopharyngeal opening exercises and functional electrical stimulation therapy. After 3 months of follow-up, severe cricopharyngeal achalasia and decreased pharyngeal contraction were also observed on VFSS.

## 3. Discussion

Cricopharyngeal achalasia refers to a state of elevated cricopharyngeal muscle or upper esophageal sphincter tone.^[3]^ Myotonic dystrophy is a neurological condition that can cause progressive cricopharyngeal dysfunction, the symptoms of which include difficulty initiating swallowing, as well as choking, regurgitation, and aspiration. In contrast to our findings, Modolell et al reported lower than average basal pressure of the upper esophageal sphincter in DM-1 patients.^[3]^ While some studies have reported lower esophageal achalasia, none have reported upper esophageal achalasia after GA in DM-1 patients.^[4,5]^

Increased anesthetic risk in the DM-1 population has been observed for a long time, and proper perianesthetic care has been emphasized.^[2]^ Hypothermia-induced shivering can induce myotonic contracture. In our case, the patient’s perioperative body temperature was maintained at 36.0 to 36.1 ºC. According to the guidelines, warm IV fluids or forced air blankets can maintain body temperature in DM-1 patients.^[6]^ Generally, DM-1 patients are sensitive to sedative-hypnotics such as propofol or opioids and require a lower doses for anesthesia.^[7]^ Opioids and hypnotics induce muscle myotonia in DM-1 patients, but the exact mechanism of cricopharyngeal tone elevation has not been determined.^[2,8]^ In the present case, the use of opioids such as remifentanil and hypnotics like propofol might have caused focal myotonic contraction on the cricopharyngeal muscle.^[2,7,9]^ The patient used rocuronium—a non-depolarizing neuromuscular blocker—and suggamadex, which reverses neuromuscular blockade induced by rocuronium, which were known to be relatively safe in DM-1 patients.^[6]^ Low body temperature, propofol, and remifentanil may aggravate myotonia, resulting in a severe cricopharyngeal achalasia pattern in DM-1.

## 4. Conclusion

We have reported a rare case of DM-1 with dysphagia aggravation and a severe cricopharyngeal achalasia pattern after GA. Monitoring and managing perioperative adverse events are essential in DM-1 patients after GA. Hypothermia-induced shivering, specific muscle relaxants, hypnotics, and opioids should be properly controlled to prevent myotonic aggravation in DM-1 patients. To our knowledge, this is the first report of cricopharyngeal achalasia aggravation proven by VFSS after GA in a DM-1 patient.

## Acknowledgments

This manuscript acquired the editorial certificate from “Editage” by Cactus (https://online.editage.co.kr/).

## Author contributions

**Conceptualization:** Yunsoo Soh.

**Investigation:** Myung Chul Yoo, Ga Yang Shim, Sung Joon Chung.

**Resources:** Yunjung Rho, Yunsoo Soh.

**Visualization:** Jinmann Chon.

**Writing – original draft:** Yunjung Rho, Yunsoo Soh.

**Writing – review & editing:** Yunjung Rho, Yunsoo Soh.
